# Teaching Professionalism to Medical Students Using Dissection-Based Anatomy Education: a Practical Guide

**DOI:** 10.1007/s40670-020-01137-2

**Published:** 2020-10-30

**Authors:** Emilia G. Palmer, Rohin K. Reddy, William Laughey

**Affiliations:** Hull York Medical School, University of York, John Hughlings Jackson Building, University Road, Heslington, York, YO10 5DD UK

**Keywords:** Education, Medical, Professionalism, Anatomy, Students, Medical, Teaching

## Abstract

Professionalism is a core competency for all healthcare professionals and is a subject of great interest within the academic community due to its vital importance in delivering the highest quality patient care. Despite this, professionalism remains difficult to define, teach and assess. The potential use of anatomy education in teaching professionalism has been increasingly highlighted within the literature, but still remains an underutilised tool in medical education. Therefore, this practical guide offers evidence-based practical points for successfully incorporating professionalism within a dissection-based anatomy course delivered to undergraduate medical students.

## Introduction

Professionalism is broadly considered to be a set of values that underpin the public’s trust in medical students and physicians [1]. Professionalism is one of the core competencies for physicians and is integral for good-quality patient care; despite this, it is often overlooked within core medical education. No single agreed definition of professionalism exists, partly due to the meaning of professionalism varying across time, contexts and cultures. Furthermore, professionalism is difficult to quantify, and thus measure [2–4]. Recently, public interest in professionalism has surged due to dissatisfaction with the performance of healthcare, development of Internet-based technology and better-educated patients. Additionally, healthcare has witnessed an increase in politicisation due to the entry of the corporate sector, altering the traditional social contract of professionalism [5–7]. Despite this, the most common reason for disciplinary action against physicians is unprofessional conduct [8], with poor professional behaviour in physicians associated with unprofessional conduct at medical school [9]. This suggests that there is significant room for improvement in how we teach, assess and remediate medical students’ professionalism. Although it is acknowledged that professional values must be taught explicitly, there is still no universally accepted practical model for doing so. Contemporary approaches typically focus on teaching the cognitive base of professionalism, which comprises its nature, principles and foundations [4]. Commonly used methods for accomplishing this include didactic lectures, case scenarios, reflective practice and role modelling [1, 5]. Growing literature has identified the utility of dissection-based anatomy education in teaching professionalism within medical education [4, 8, 10–12]. Firstly, as dissection-based anatomy education is a core component of many preclinical medical curricula, it is a perfect, early opportunity to begin shaping professional identities [2, 13]. Additionally, anatomy faculty have the opportunity to exhibit many key attributes of professionalism, representing key, positive role models for students [2]. Furthermore, within dissection-based anatomy education, the cadaver, often embodying the students’ ‘first patient’, represents a powerful tool for instilling professional values, such as respect, dignity and integrity, whilst developing students’ coping skills [14]. Lastly, within the dissection room, students have the opportunity to regularly work within a group, instilling the skills of teamwork, independent thinking, accountability and leadership [2, 15–17].

Clearly, dissection-based anatomy education is an ideal setting for teaching basic elements of professionalism and is currently a neglected opportunity. Considering that many medical institutions use dissection for teaching anatomy to medical students, this practical, evidence-based guide is relevant to many educators who wish to incorporate professionalism teaching within their medical curricula. It is specifically aimed at providing guidance for educators within medical curricula that teach anatomy to preclinical students using dissection.

### Agree on a Definition of Professionalism in Order to Create Learning Objectives

**Fig. 1:**
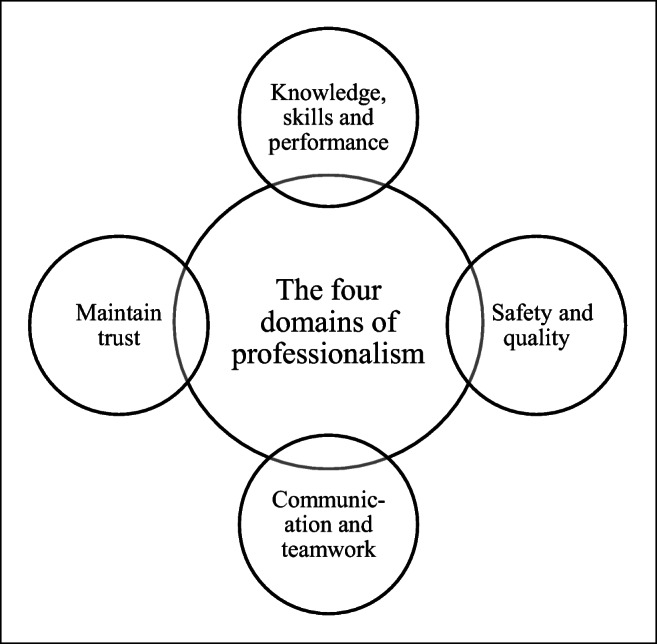
The four domains of professionalism, adapted from the GMC [18] and Van De Camp et al. [19]

As a unanimous definition of professionalism does not exist, the anatomy faculty must reach a consensus on what professionalism means to them [2]. Synthesising the definitions created by the General Medical Council (GMC), a public body that maintains an official register of medical practitioners within the UK [18], and Van De Camp and colleagues’ systematic review [19], a potential definition of professionalism specific to medical students undertaking dissection-based anatomy education has been created. This definition has been divided into four domains of professionalism, as depicted in Fig. 1. The first domain is knowledge, skills and performance, which encompasses the need of students to be competent in both anatomical and professional knowledge, whilst also committing to lifelong learning. The second is safety and quality, which refers to respect towards cadavers, and donning correct protective equipment whilst in the dissection room. The third is communication and teamwork, which includes good teamwork, integrity, providing feedback and developing communication skills. The last is to maintain trust, by upholding not only the trust society has granted through allowing students to dissect cadavers, but also the responsibility and respect the students must demonstrate towards the cadaver, who often represents students’ first patient.

This definition should guide the formation of learning objectives, which outline clear observable behaviours expected of students. These learning objectives form the cognitive base of professionalism, outlining which concepts will be taught, evaluated and assessed [1, 4]. Learning objectives should be easily available for reference, i.e. within virtual learning environments or the student handbook in order to remind students of the professional behaviours expected from them [2, 8, 20]. In Table 1, example learning objectives have been created based on the work of Ghosh [11] and GMC guidelines [18].

**Table 1:** Example learning objectives, adapted from Ghosh [11] and GMC guidelines [18]

• Keep up to date in all aspects of your studies • Review past learning with the aim of improvement • Take prompt action if you believe a colleague is acting unprofessionally towards a cadaver • Treat colleagues with respect at all times • Acknowledge and reflect on personal unconscious biases • Always treat cadavers with the upmost of respect – this includes upholding their confidentiality, integrity and acknowledgment that the cadaver once constituted human life • Always adorn the correct personal protective equipment • Take care to keep all tissues with the cadaver • Attend the memorial service • Be honest if a mistake has been made	

### Recognise the Value of a Good First Impression

Dissection has been found to be an emotional and often stressful experience for many students [21]. Despite this, through regularly working with a cadaver, the students are encouraged to develop healthy coping mechanisms for dealing with death [2, 11, 22]. To reduce the stress associated with dissection, whilst promoting the acquisition of positive coping mechanisms, the faculty should provide an initial dissection demonstration which sensitively introduces students to both the cadaver and dissection [23]. Within this session, faculty should accommodate and provide validation for the wide range of emotions students may experience during dissection, including both emotional distress and detachment [14]. Furthermore, it is crucial that the faculty model key professional values during this session, including respect and sensitivity to the cadaver, encouraging students to exhibit the same values and behaviours. In addition to this, the faculty must remain sensitive to students’ emotions around death and dissection and provide appropriate student support services [2, 7].

### Identifying the Donor

Currently, no consensus exists on whether the identity of the cadaver should be revealed to students. A majority of curricula choose to maintain full confidentiality of the cadaver, whilst others reveal varying amounts of the donor’s details [24]. Within curricula maintaining donor anonymity, students have been noted to adopt the practice of ‘cadaver naming’, whereby they create unique nicknames for donors, often based on the donor’s age or notable physical attributes [25, 26]. This may help students better cope with emotional stresses of dissection, especially valuable within the early stages, whilst students adjust to death. However, cadaver naming may result in emotional distance between the physical body and the complex humanity it once represented. Thus, during times of emotional stress, these future physicians may revert to naming, and thus reducing, a patient to merely their condition or unwell organ, compromising a holistic view of the patient [26]. Furthermore, students faced with donor anonymity may question the donors’ wishes for dissection of their body, and therefore out of respect for the donor, feel uncomfortable, or even decline performing certain dissections, such as forceful acts [14]. Donors, however, do not perceive conflict between dissection of their body and exhibiting respect [27]. Thus, the disconnect between students’ and donors’ views of respect can lead to students’ unnecessarily experiencing distress and missing learning opportunities [14].

Some medical schools have instead chosen to reveal the identity of the donor to students, through revealing donors’ birth names or showing students prerecorded videos of the donor sharing details of themselves, their condition and reasons for donation [14, 26, 28]. This encourages perception of the cadaver as a human being, rather than simply a teaching tool, and facilitates student comprehension of the honour of dissection. Students are often eager to gain more details about the donor, which may facilitate closer student-donor bonds [14, 26, 28]. This teaches students crucial professional values including empathy, confidentiality and respect, and encourages deeper contemplation of the ethical questions surrounding dissection [14, 24, 26, 29]. Additionally, through revealing the donor’s wishes for donation and their understanding of the nature of dissection, students’ concern about carrying out potentially disrespectful acts of dissection will be eased [14]. However, revealing donor identities will create important confidentiality considerations for both faculty and students, which must be addressed through careful planning, support and education [29].

A limited evidence base precludes a definitive best practice approach, and therefore faculty must consider the theorised benefits and drawbacks of each method in order to decide which approach best suits their goals and values. However, we suggest that although it may not be the current norm to reveal the identity of the donor, anatomy educators should carefully consider the potential benefits this may have on students’ professional identities [29].

### Understand Your Responsibilities as a Role Model

Role modelling has been identified as the most effective method for teaching professionalism across multiple studies [5, 30, 31]. Students have been found to be constantly observing the behaviours of their role models, demonstrating the power they have in positively shaping students’ professional values, and ultimately identities [4, 32]. Despite this, role models have been demonstrated to frequently display lapses in professional behaviour, exhibiting unprofessional conduct [33, 34]. The witnessing of unprofessional conduct is often difficult for students to process, resulting in internal conflict within those who would otherwise possess positive professional values [2, 35]. Furthermore, students witnessing unprofessional conduct has been linked to the learning of negative attitudes, adopting emotional neutralisation, cynicism and demotivation [33, 36].

Tutor education is key to enhancing positive role modelling and reducing the frequency of students witnessing unprofessional conduct. Anatomy educators interact with their students in a multitude of situations, including but not limited to dissection sessions, seminars and lectures. The faculty must understand that their behaviours, attitudes and values are constantly shaping students’ professional identities, and thus must continually display the same professional behaviours expected from students, regardless of the situation [4, 32]. In particular within the dissection room, educators must be acutely aware of the nature in which they discuss cadavers, with a particular emphasis on upholding their confidentiality and dignity, whilst acknowledging that the cadaver once constituted human life. Furthermore, educators should consider the manner in which they teach anatomy within seminars and lectures, and the way they interact with both students and other members of the faculty. To encourage accountability, a culture in which students feel comfortable reporting unprofessional conduct must be fostered by the faculty, such as through the use of an anonymous reporting system.

### Address the Hidden Curriculum

The hidden curriculum encompasses the learning experiences that occur outside of formal education [2, 37]. The hidden curriculum includes the unplanned interactions students have with faculty, and the culture created by the faculty, alongside the organisational infrastructure [1, 7]. The hidden curriculum plays a significant role in formation of students’ professional identities and exerts both a positive and/or negative influence on students [4, 6, 36]. Specific to dissection-based anatomy education, the hidden curriculum exerts its influence during the interactions students have with faculty when walking from the seminar/lecture room to the dissection laboratory, during coffee breaks, and when adorning personal protective equipment. In order to reduce the frequency of students witnessing unprofessional conduct during these interactions, the faculty should be educated regularly around the importance of continuously modelling professional values whilst at work. Although less immediately apparent, the culture created by the anatomy faculty can have a strong impact on student’s professional values. To promote a positive culture, reflective work should be incorporated into faculty education such that faculty address their own negative professional values and/or biases. Additionally, through implementing a bottom-up approach in which students are encouraged to report faculty who display unprofessional conduct, a culture of local resolution and accountability, as described by Al-Eraky, will be encouraged [4].

### Utilise the Cadaver as a Tool for Teaching Professionalism

Within dissection-based anatomy education, the students will spend a considerable proportion of their time working in a group, dissecting a cadaver within the laboratory. It is there that the students begin to develop values from all domains of professionalism. Due to this, the cadaver represents one of the most important tools within a dissection-based anatomy curriculum for shaping the student’s professional identities [22].

Through representing the students’ first patient, the manner in which the students treat the cadaver can shape their future interactions with patients. Due to this, the faculty, as important role models, must be encouraged to consistently model values from all four domains of professionalism, including demonstrating their commitment to anatomical learning; wearing personal protective equipment at all times; working well with colleagues and other students; and lastly, showing respect towards the cadaver [2, 4]. Additionally, as the cadaver cannot defend itself, it signifies the extreme dependence of a patient on his/her doctor, posing additional moral and professional challenges for the students [14]. Without any objections from the cadaver, the students must make important moral decisions, including what language they use when referring to the cadaver, whether they keep all the tissue with the body and whether they cover the cadaver’s face and genitals [22]. Having to answer these moral questions encourages students, early on in their medical education, to learn important professional values that will be directly applicable to their future practice as doctors. These include maintaining the confidentiality and dignity of patients and behaving with integrity and respect [18]. Early exposure to these concepts during preclinical years will encourage lifelong consideration of their importance. Within the introduction session, the faculty should encourage students to feel comfortable reporting any colleagues demonstrating unprofessional behaviour, as an additional measure for monitoring students during these influential formative experiences of professionalism. Additionally, there should always be a member of staff within the dissection room observing the students in order to ensure they are practicing to the highest professional standards.

### Use Case Scenarios to Teach the Cognitive Base

Many advocate teaching the cognitive base of professionalism [4, 6, 20], with case scenarios evidenced as an effective [30, 31] and enjoyable method for doing so [38]. The use of case scenarios is further justified by empirical literature studying problem-based learning which is grounded in the use of case scenarios to facilitate learning. Thus, literature supporting problem-based learning may be applied to the use of case scenarios within anatomy education. Two independent meta-analyses [39, 40] found that problem-based learning teaches students to apply their knowledge more effectively than traditional learning approaches, such as didactic lectures. Furthermore, problem-based learning is perceived by students as more enjoyable, whilst simultaneously teaching students key professional skills such as teamwork and communication [41–43]. The effectiveness of case scenarios is grounded within the situated learning theory, which postulates that learning is most successful when students are taught the cognitive base and are subsequently provided with opportunities to utilise this new knowledge in an authentic context, emphasising its usefulness [1, 6].

Guided by the aforementioned literature, case scenarios should be incorporated into the curriculum to facilitate learning of the cognitive base of professionalism. Weekly seminar-based case scenarios should focus on thought-provoking topics or simulated encounters, triggering discussion within a safe environment and allowing an authentic context for learning [30, 38, 44]. Within these case scenarios, the facilitator must prompt the students to explore key areas of the cognitive base outlined within the learning objectives, including but not limited to the following: the origins of professionalism, its characteristics, the importance it holds and the behaviours necessary to sustain it [1, 6]. An example case scenario and important areas for discussion within this seminar have been outlined within Table 2. Additionally, the curriculum should include a seminar focused on the ethics of dissection, allowing students to explore both the ethical and humanitarian aspects of dissecting cadavers. This seminar may foster professional values including respect towards the cadaver, whilst allowing students to begin to comprehend the great privilege and social responsibility afforded to them by dissection [12].

**Table 2:** An example case scenario, with areas for discussion

Case scenario:	Areas for discussion:
During a dissection session, a student notices their colleagues are behaving disrespectfully to the cadaver. They are using inappropriate names, laughing at the donor’s weight and not taking appropriate care with dissection.	• Are the students behaving in a professional manner? If not, which values are they undermining? • What actions could the observing student take to uphold the values of professionalism? • Why is behaving in a professional manner important within the dissection room? • How would the donor feel about the way their body, donated for the advancement of medical science, was being treated? • How would the donor’s family members feel if they knew their family member was being treated in that way?

### Encourage Teamwork

The dissection room is an ideal setting for incorporating teamwork and communication skills into the curriculum, therefore teaching the third domain of professionalism. On a termly basis, assign students into groups of four; within this group, they are required to work together dissecting the cadaver and completing relevant learning objectives. This not only promotes learning of critical professional skills, such as language, respect and integrity, but also allows students to understand the importance of working within a group accountable for all members’ learning, promoting responsibility [2, 23]. The use of predefined roles allows the genuine sharing of responsibilities, promotes accountability and reduces inactivity of team members less inclined to contribute [8]. Adapting and incorporating the recommendations of Escobar-Poni and Poni [8] and Heyns [15], alongside our own experience, four distinct roles have been defined, as outlined in Table 3.

**Table 3:** Responsibilities of each role within groups in the dissection room [8, 15]

Role	Responsibilities
Dissector	Performs the physical dissection.
Assistant dissector	Assists the main dissector in dissection tasks, maintains the work area and equipment.
Reader	Aids in facilitating correct dissection through referring to the atlas, dissection instructions and facilitators.
Scribe	Relates the anatomy to the learning objectives and clinical cases. Photographs the cadaver.

Teamwork may also be promoted within student-led seminars orientated around the weekly dissection, facilitating development of professional skills such as public speaking, leadership and scientific knowledge [8, 23, 45]. Thus, learning to work well in a team is both a professional responsibility and an effective way for students to develop other crucial professional skills.

### Explore the Use of Online Resources

The use of online resources, such as an app, wiki forum or social media platform (e.g. Facebook), to teach professionalism has recently received attention within published literature [4, 46, 47]. Ninety per cent of students use social media, and 50% blogs or wikis, suggesting that these online resources represent a powerful and often neglected tool for implementing positive professional values in students. The use of small-group wikis as a space to share resources relating to the student’s professionalism learning objectives was found to foster positive group dynamics, create a shared knowledge base, encourage reflection on the quality of resources and lastly support the development of professional identities [46]. Additionally, Facebook has been found to be an effective and enjoyable platform for students to reflect and receive feedback on professional behaviours they witnessed during clinical practice [47].

Online resources should be incorporated within the anatomy curriculum based on their proven power in promoting professionalism. This could be done through the use of bespoke apps or Facebook in order to create a safe space for students to reflect and receive feedback on professional behaviours they have observed from colleagues or educators within the formal or hidden anatomy curriculum. Through receiving feedback, the students are encouraged to identify whether the behaviour they witnessed was positive or negative, reducing the potential detrimental effect of students witnessing unprofessional conduct and developing the students’ professional identities [48]. Alternatively, wikis can be utilised within a group project aiming to foster teamwork, self-directed learning and commitment to excellence. The faculty should assign students into groups of approximately five: within this group, they are required to create a wiki outlining an anatomical topic chosen by either the students or faculty [46, 47]. Using one or both of these online resources, students will learn key anatomical concepts, alongside developing their professional behaviours in a collaborative, engaging and innovative manner that will benefit their future practice.

### Promote Continuous Reflection

Reflection is a key tool for teaching professionalism, and has been shown to reduce the effect of negative role models and experiences [7, 49]. Reflection is linked to multiple core professional values including critical thinking, integration of theoretical concepts and empathy. Furthermore, physicians’ ability to reflect has been linked to improvement of patient care, most likely because reflection serves as a tool for self-improvement and identification of errors [2, 23, 50].

Anatomy education could serve as an early opportunity for incorporating reflective practice within undergraduate medical students, who may have limited experience in this field. One key area of reflection within anatomy education is the memorial service, a day held to respect and honour those who donated their body to medical science [23]. The memorial service is an important day for shaping the student’s professional identities and developing important professional values. The service reinforces the student’s feelings of respect and compassion towards the cadaver, as defined within the second domain of professionalism. Additionally, the service allows students to comprehend the privilege the donation afforded them and encourages the students to act professionally within the dissection room in order to uphold the trust the donor, and society as a whole, has afforded them, as defined within domain 4 of professionalism. Furthermore, the service provides closure for families, an important element of their grieving process that may be overlooked by students [2, 8].

In order to encourage continuous reflection, the faculty should provide regular compulsory opportunities for students to reflect, such as writing weekly reflective passages around development of their professional identities. Passages should include what they have learned, progress, identification of weaknesses, future targets and exploration of any difficult emotions they have experienced within the dissection laboratory [14, 45].

### Assess Professionalism

Continuous assessment of students’ professional development allows the faculty to measure students’ achievement of their professionalism learning objectives, and facilitates provision of feedback, acting as a driver for student improvement [1, 6]. Despite this, there has been debate as to whether professionalism can be assessed adequately and reliably [7]. It has been concluded that no single tool is able to reliably and effectively measure students’ professionalism, and instead a multi-tool approach should be used due to the complex and situational nature of professionalism [7, 51]. Furthermore, this approach reduces the impact of individual tools’ limitations and, through capturing students in different situations, reduces the risk of students ‘staging’ professional behaviours [3, 5]. Based on this, this paper advocates a three-tier approach using peer assessment, progress notes and a student portfolio. It is important to explain to students early that professionalism will be assessed continuously in order to drive their learning and development, encouraging students to view professionalism as important and positive [3].

#### Peer Assessment

Peer assessment has been found to be effective in monitoring students’ professional values and improving professionalism; therefore, it should be at the forefront of the assessment process [52–55]. Within a dissection-based anatomy education, the students will be regularly and consistently working within a group; and therefore, it represents an excellent opportunity for the incorporation of peer assessment. Peer assessment can be incorporated through requiring students to provide termly anonymous feedback to other members of both their dissection and wiki groups. This may be performed via online peer-rating forms including positive and negative stems around professional behaviours such as communication, punctuality, respect for peers and faculty, and commitment to excellence [45]. The survey should consist of a mix of 5-point Likert scale and open questions, to facilitate further explanation.

Peer assessment allows for early identification of areas in which professional behaviour could be improved. Additionally, when students provide feedback for others, they reflect and gain insight into their own behaviours [8, 10]. Peer assessment is best used formatively as students are less likely to provide honest, critical feedback to peers if it jeopardises their grades [3, 20, 54], and because some students, especially those in earlier years of training, may struggle providing reliable and justified peer assessments [56]. However, faculty may choose to summatively assess the quality of feedback the students provide, such as through a validated measurement tool [57].

#### Progress Notes

Progress notes are weekly passages students are required to write so that the faculty can monitor their overall academic progress. The students should answer questions such as ‘what have I learnt?’, ‘how did I learn?’, ‘are there gaps in my knowledge or skills?’ and ‘are there areas for my academic or professional development?’. Progress notes should thus encourage the learning of responsibility, independence and continued commitment to excellence, all key professional skills that are important for future physicians to develop early and maintain throughout their careers [8].

#### Student Portfolio

The results of students’ peer assessment, alongside their progress notes and any critical incident reports, should be collated within a student portfolio, which will display the students’ progress, and allow tutors to provide one-on-one, individual termly feedback [5, 45, 55]. This would provide the framework for an open and honest discussion regarding the students’ specific behaviours and attitudes, highlighting clear and tangible individual areas for improvement [4].

### Remedy Lapses in Professionalism Quickly and Fairly

Lapses in students’ professional behaviour during medical school have been linked to later disciplinary action [9]; therefore, lapses in professional values must be addressed quickly and effectively to prevent subsequent, avoidable harm to patients, colleagues or their future student mentees [23]. Within the dissection room, as students become accustomed to death, whilst simultaneously dissecting deeper within the cadaver, it can be increasingly easy to forget the humanity the cadaver once represented, resulting in some students forgetting their professional responsibilities within the dissection room [14, 22, 58]. Through encouraging the students to report any unprofessional conduct they observe, ensuring a faculty member is always within the dissection room and assessing professionalism at regular intervals, students displaying unprofessional conduct or attitudes are identified quickly. These students should be invited to meetings with faculty members which incorporate three key elements, as described by Altirkawi [7], namely, education around the meaning of professionalism, its importance and behaviours encompassed within its values; suggestions on how the student can amend unprofessional conduct; and finally, explicit explanation of subsequent consequences if the student fails to improve their conduct. The student should be monitored, and follow-up scheduled in order to praise improvement and identify non-improvers [20].

### Evaluate for Professionalism

The most detailed policies and the best intentions will count for nothing if those delivering anatomy education exhibit anything less than the highest professional standards. Evaluation and feedback are key methods for ensuring adherence to the highest professional standards whilst encouraging lifelong, continual improvement in this area [59]. One tool for measuring educators’ professional behaviour is through the use of student feedback. This can be obtained through questions measuring specific behaviours of the faculty member, practically this may be done through an app or an online or paper-based questionnaire. Furthermore, a culture in which students feel comfortable reporting unprofessional conduct must be fostered [4]. An additional tool for measuring educators’ professionalism within teaching includes peer observation [60]. This is especially important for new faculty members, such as anatomy demonstrators who specifically may have relatively brief teaching tenures. Lastly, faculty members who receive positive student feedback, or are otherwise acknowledged for their dedication to professional values, must be rewarded, for example within an annual award ceremony to foster and encourage excellence within professionalism education [61]. This would have the additional benefit of fostering a community of professionalism within the anatomy faculty, which the students would have had an active part in promoting, increasing their own sense of professional pride.

## Conclusion

Anatomy education serves as an excellent opportunity for incorporation of professional values and behaviours within medical students at early stages of their career. In modern healthcare systems which increasingly require physicians to display positive professional behaviours, this opportunity is even more valuable. Anatomy educators must consider what professionalism means to them, and subsequently create learning objectives outlining the cognitive base expected from students. In order to successfully integrate positive professional values, faculty must be aware of their powerful position as role models, and thus must consistently act professionally. Furthermore, faculty must utilise important tools for instilling professional values, including teamwork, reflective practice and explicit teaching of professionalism through the use of case scenarios and online resources. As the cadaver represents the altruistic donation society makes to medical education, it can be used to emphasise key aspects of professionalism whilst providing a practical model for the students to apply the concepts they have learned from the anatomy faculty. Lastly, a multi-tool approach to assess professionalism must be utilised, and the behaviours of educators evaluated. The thirteen practical points and approaches for implementing them have been summarised within Table 4.

**Table 4:** A summary of the practical points, alongside approaches for their implementation

Points	Practical approaches for implementation
Agree on a definition of professionalism in order to create learning objectives	• Each faculty must create their own definition of professionalism. • Guided by this definition, learning objectives should clearly outline which concepts of professionalism will be taught and assessed.
Recognise the value of a good first impression	• Educators must sensitively introduce students to the cadaver whilst displaying professional behaviours within an initial dissection session. • Throughout the course, educators must remain sensitive to students’ feelings about death and dissection and provide appropriate student support services.
Donor identification	• Individual faculties must decide whether they reveal the donor’s identity, guided by the theorised benefits and drawbacks of each approach.
Understand your responsibilities as a role model	• Educate tutors on the importance of modelling good professional values. • Encourage and enable students to feel comfortable reporting any unprofessional conduct they have witnessed.
Address the hidden curriculum	• To reduce the potential negative effects of the hidden curriculum: • Educate faculty about the necessity of always modelling professional values, regardless of the situation. • Incorporate reflective work into faculty education. • Encourage students to report faculty who display unprofessional conduct.
Utilise the cadaver as a tool for teaching professionalism	• Within the dissection room, faculty must model values from all domains of professionalism, including respect towards the cadaver. • Urge students to report colleagues behaving unprofessionally towards the cadaver. • A member of staff should always observe the student’s behaviour within the dissection room.
Use case scenarios to teach the cognitive base	• Teach the cognitive base of professionalism through weekly case scenarios, delivered within seminars. • One seminar should focus on the ethics of dissection.
Encourage teamwork	• Encourage teamwork through: - Assigning students into small groups within the dissection room. Within this group students should be allocated a pre-defined role. - Student-led seminars discussing the weekly dissection.
Explore the use of online resources	• Online resources can be utilised to: - Create a space in which students and faculty can discuss professional behaviours students have observed. - Facilitate a group project, such as the creation of a wiki.
Promote continuous reflection	• The memorial service is important for developing students’ professional values. • Students should complete weekly reflective passages, including what they have learnt, their progress, identification of weaknesses and future targets.
Assess professionalism	• Assess students’ professionalism through a multi-tool approach, this could include: - Peer assessment: Encourage students to provide termly, anonymous feedback to other members of their dissection and/or wiki group. - Progress notes: Students should write weekly passages outlining their academic progress. - Student portfolio: Within a portfolio, collate students’ peer assessment results, progress notes and any critical incident reports, in order to guide one-on-one, individual tutor feedback.
Remedy lapses in professionalism quickly and fairly	• Students exhibiting unprofessional conduct must be identified quickly; these students should be scheduled a meeting with a faculty member, monitored and followed-up.
Evaluate for professionalism	• Encourage students to provide feedback on the professional behaviours of faculty members. • Foster a culture in which students are comfortable reporting unprofessional conduct. • Incorporate peer observation for anatomy educators. • Reward educators displaying professional values within an annual award ceremony.

Due to the ongoing COVID-19 pandemic, many institutions have adopted strategies to deliver medical education online. Consequently, there is uncertainty regarding when, and even whether, dissection- and prosection-based anatomy education will recommence [62]. In light of this, professionalism teaching must be incorporated into online anatomical education. This could be achieved through adapting previously in-person case-scenario sessions and student-led seminars into online platforms or video conferencing software. Online, weekly reflection and progress notes will serve as key tools for both encouraging and assessing the development of students’ professional behaviours. Tools for remotely teaching gross anatomy, such as ‘Acland’s video atlas of human anatomy’ [63], an online bank of real human anatomical specimens, are available to medical institutions. However, faculty must continue to remain sensitive to the complex range of emotions students may feel when confronted with cadavers, even via online platforms, and provide appropriate student support services. Furthermore, previously suggested methods of teaching professionalism online, including group wikis and social media, will assume even greater importance during the present time of online medical education.

## Data Availability

Not applicable

## References

[1] O’Sullivan H, van Mook W, Fewtrell R, Wass V (2012). Integrating professionalism into the curriculum: AMEE guide no. 61. Med Teach.

[2] Swick HM (2006). Medical professionalism and the clinical anatomist. Clin Anat.

[3] Goldie J (2013). Assessment of professionalism: a consolidation of current thinking. Med Teach.

[4] Al-Eraky MM (2015). Twelve tips for teaching medical professionalism at all levels of medical education. Med Teach.

[5] Morihara SK, Jackson DS, Chun MB (2013). Making the professionalism curriculum for undergraduate medical education more relevant. Med Teach.

[6] Cruess RL, Cruess SR (2006). Teaching professionalism: general principles. Med Teach.

[7] Altirkawi K (2014). Teaching professionalism in medicine: what, why and how?. Sudan J Paediatr.

[8] Escobar-Poni B, Poni ES (2006). The role of gross anatomy in promoting professionalism: a neglected opportunity!. Clin Anat.

[9] Papadakis MA, Teherani A, Banach MA, Knettler TR, Rattner SL, Stern DT, Veloski JJ, Hodgson CS (2005). Disciplinary action by medical boards and prior behavior in medical school. N Engl J Med.

[10] Bryan RE, Krych AJ, Carmichael SW, Viggiano TR, Pawlina W (2005). Assessing professionalism in early medical education: experience with peer evaluation and self-evaluation in the gross anatomy course. Ann Acad Med Singap.

[11] Ghosh SK (2017). Paying respect to human cadavers: we owe this to the first teacher in anatomy. Ann Anat.

[12] Karunakaran I, Thirumalaikolundusubramanian P, Nalinakumari SD (2017). A preliminary survey of professionalism teaching practices in anatomy education among Indian medical colleges. Anat Sci Educ.

[13] Pawlina W, Hromanik MJ, Milanese TR, Dierkhising R, Viggiano TR, Carmichael SW (2006). Leadership and professionalism curriculum in the gross anatomy course. Ann Acad Med Singap.

[14] Goss AL, Viswanathan VB, DeLisser HM (2019). Not just a specimen: a qualitative study of emotion, morality, and professionalism in one medical school gross anatomy laboratory. Anat Sci Educ.

[15] Heyns M (2007). A strategy towards professionalism in the dissecting room. Eur J Anat.

[16] Pearson WG, Hoagland TM (2010). Measuring change in professionalism attitudes during the gross anatomy course. Anat Sci Educ.

[17] Lempp HK (2005). Perceptions of dissection by students in one medical school: beyond learning about anatomy. A qualitative study. Med Educ.

[18] General Medical Council. Good medical practice. 2013. https://www.gmc-uk.org/-/media/documents/good-medical-practice%2D%2D-english-20200128_pdf-51527435.pdf?la=en&hash=DA1263358CCA88F298785FE2BD7610EB4EE9A530. Accessed 8 Apr 2020.

[19] Van De Camp K, Vernooij-Dassen MJ, Grol RP, Bottema BJ (2004). How to conceptualize professionalism: a qualitative study. Med Teach.

[20] Macneill P, Joseph R, Lysaght T, Samarasekera DD, Hooi SC (2020). A professionalism program in medical education and training - from broad values to specific applications: YLL School of Medicine, Singapore. Med Teach.

[21] Nnodim JO (1996). Preclinical student reactions to dissection, death, and dying. Clin Anat.

[22] Kermanian F, Farajidana H, Motaharipour M (2018). Professionalism in clinical anatomy based on cadaver dissection: the importance of principles and ideals. Int J Anat Res.

[23] Sugand K, Abrahams P, Khurana A (2010). The anatomy of anatomy: a review for its modernization. Anat Sci Educ.

[24] Talarico EF (2013). A change in paradigm: giving back identity to donors in the anatomy laboratory. Clin Anat.

[25] Williams AD, Greenwald EE, Soricelli RL, DePace DM (2014). Medical students' reactions to anatomic dissection and the phenomenon of cadaver naming. Anat Sci Educ.

[26] Kumar Ghosh S, Kumar A (2019). Building professionalism in human dissection room as a component of hidden curriculum delivery: a systematic review of good practices. Anat Sci Educ.

[27] Olejaz M, Hoeyer K (2016). Meet the donors: a qualitative analysis of what donation means to Danish whole body donors. Eur J Anat.

[28] Bohl M, Holman A, Mueller DA, Gruppen LD, Hildebrandt S (2013). The willed body donor interview project: medical student and donor expectations. Anat Sci Educ.

[29] Jones DG, King MR. Maintaining the anonymity of cadavers in medical education: historic relic or educational and ethical necessity? Anat Sci Educ. 2017;10(1). 10.1002/ase.1618. 10.1002/ase.1618 27123986

[30] Byszewski A, Hendelman W, McGuinty C, Moineau G (2012). Wanted: role models--medical students’ perceptions of professionalism. BMC Med Educ.

[31] Roberts LW, Green Hammond KA, Geppert CM, Warner TD (2004). The positive role of professionalism and ethics training in medical education: a comparison of medical student and resident perspectives. Acad Psychiatry.

[32] Finn, Garner J, Sawdon M (2010). ‘You’re judged all the time!’ Students’ views on professionalism: a multicentre study. Med Educ.

[33] Lempp H, Seale C (2004). The hidden curriculum in undergraduate medical education: qualitative study of medical students’ perceptions of teaching. BMJ..

[34] Wear D, Zarconi J (2008). Can compassion be taught? Let’s ask our students. J Gen Intern Med.

[35] Brainard AH, Brislen HC (2007). Viewpoint: learning professionalism: a view from the trenches. Acad Med.

[36] Karnieli-Miller O, Vu TR, Frankel RM, Holtman MC, Clyman SG, Hui SL, Inui TS (2011). Which experiences in the hidden curriculum teach students about professionalism?. Acad Med.

[37] Karnieli-Miller O, Vu TR, Holtman MC, Clyman SG, Inui TS (2010). Medical students’ professionalism narratives: a window on the informal and hidden curriculum. Acad Med.

[38] Spampinato CM, Wittich CM, Beckman TJ, Cha SS, Pawlina W (2014). “Safe Harbor”: evaluation of a professionalism case discussion intervention for the gross anatomy course. Anat Sci Educ.

[39] Dochy F, Segers M, Van den Bossche P, Gijbels D (2003). Effects of problem-based learning: a meta-analysis. Learn Instr.

[40] Kasim RM (1999). What can studies of problem-based learning tell us? Synthesizing and modeling PBL effects on National Board of Medical Examination Performance: hierarchical linear modeling meta-analytic approach. Adv Health Sci Educ Theory Pract.

[41] Albanese MA, Mitchell S (1993). Problem-based learning: a review of literature on its outcomes and implementation issues. Acad Med.

[42] Vernon DT, Blake RL (1993). Does problem-based learning work? A meta-analysis of evaluative research. Acad Med.

[43] Colliver JA (2000). Effectiveness of problem-based learning curricula: research and theory. Acad Med.

[44] Hill-Sakurai LE, Lee CA, Schickedanz A, Maa J, Lai CJ (2008). A professional development course for the clinical clerkships: developing a student-centered curriculum. J Gen Intern Med.

[45] Elliott DD, May W, Schaff PB, Nyquist JG, Trial J, Reilly JM, Lattore P (2009). Shaping professionalism in pre-clinical medical students: professionalism and the practice of medicine. Med Teach.

[46] Varga-Atkins T, Dangerfield P, Brigden D (2010). Developing professionalism through the use of wikis: a study with first-year undergraduate medical students. Med Teach.

[47] Hsieh JG, Kuo LC, Wang YW. Learning medical professionalism – the application of appreciative inquiry and social media. Med Educ Online. 2019;24(1). 10.1080/10872981.2019.1586507. 10.1080/10872981.2019.1586507 PMC6407573 30831060

[48] Peters D, Horn C, Gishen F (2018). Ensuring our future doctors are resilient. BMJ..

[49] Stephenson AE, Adshead LE, Higgs RH (2006). The teaching of professional attitudes within UK medical schools: reported difficulties and good practice. Med Educ.

[50] Lachman N, Pawlina W (2006). Integrating professionalism in early medical education: the theory and application of reflective practice in the anatomy curriculum. Clin Anat.

[51] Wilkinson TJ, Wade WB, Knock LD (2009). A blueprint to assess professionalism: results of a systematic review. Acad Med.

[52] Schonrock-Adema J, Heijne-Penninga M, van Duijn MA, Geertsma J, Cohen-Schotanus J (2007). Assessment of professional behaviour in undergraduate medical education: peer assessment enhances performance. Med Educ.

[53] Spandorfer J, Puklus T, Rose V, Vahedi M, Collins L, Giordano C, Schmidt R, Braster C (2014). Peer assessment among first year medical students in anatomy. Anat Sci Educ.

[54] Finn, Sawdon M, Clipsham L, McLachlan J (2009). Peer estimation of lack of professionalism correlates with low conscientiousness index scores. Med Educ.

[55] Camp CL, Gregory JK, Lachman N, Chen LP, Juskewitch JE, Pawlina W (2010). Comparative efficacy of group and individual feedback in gross anatomy for promoting medical student professionalism. Anat Sci Educ.

[56] Mullikin TC, Shahi V, Grbic D, Pawlina W, Hafferty FW (2019). First year medical student peer nominations of professionalism: a methodological detective story about making sense of non-sense. Anat Sci Educ.

[57] Wittich CM, Pawlina W, Drake RL, Szostek JH, Reed DA, Lachman N, McBride JM, Mandrekar JN, Beckman TJ (2013). Validation of a method for measuring medical students’ critical reflections on professionalism in gross anatomy. Anat Sci Educ.

[58] Swartz WJ. Using gross anatomy to teach and assess professionalism in the first year of medical school. Clin Anat. 2006;19(5). 10.1002/ca.20331. 10.1002/ca.20331 16683238

[59] Kogan JR, Shea JA (2007). Course evaluation in medical education. Teach Teach Educ.

[60] Sullivan PB, Buckle A, Nicky G, Atkinson SH (2012). Peer observation of teaching as a faculty development tool. BMC Med Educ.

[61] General Medical Council. Promoting professionalism. 2020. https://www.gmc-uk.org/-/media/documents/promotingprofessionalism-dc10904_pdf-73314128.pdf. Accessed 8 Apr 2020.

[62] Iwanaga J, Loukas M, Dumont AS, Tubbs RS. A review of anatomy education during and after the COVID-19 pandemic: revisiting traditional and modern methods to achieve future innovation. Clin Anat. 2020;18:10.1002/ca.23655. 10.1002/ca.23655. 10.1002/ca.23655 PMC7404762 32681805

[63] Wolters Kluwer. Acland’s video atlas of human anatomy. https://aclandanatomy.com/. Accessed 16 Oct 2020.

